# Vertically aligned ZnO nanorod core-polypyrrole conducting polymer sheath and nanotube arrays for electrochemical supercapacitor energy storage

**DOI:** 10.1186/1556-276X-9-453

**Published:** 2014-08-31

**Authors:** Navjot Kaur Sidhu, Alok C Rastogi

**Affiliations:** 1 Electrical and Computer Engineering Department and Center for Autonomous Solar Power (CASP), Binghamton University, State University of New York, New York 13902, USA

**Keywords:** Zinc oxide nanorods, Polypyrrole nanotubes, 3-D nanostructures, Supercapacitor, Pulsed electrochemical polymerization, Electrochemical energy storage, Redox capacitance

## Abstract

Nanocomposite electrodes having three-dimensional (3-D) nanoscale architecture comprising of vertically aligned ZnO nanorod array core-polypyrrole (PPy) conducting polymer sheath and the vertical PPy nanotube arrays have been investigated for supercapacitor energy storage. The electrodes in the ZnO nanorod core-PPy sheath structure are formed by preferential nucleation and deposition of PPy layer over hydrothermally synthesized vertical ZnO nanorod array by controlled pulsed current electropolymerization of pyrrole monomer under surfactant action. The vertical PPy nanotube arrays of different tube diameter are created by selective etching of the ZnO nanorod core in ammonia solution for different periods. Cyclic voltammetry studies show high areal-specific capacitance approximately 240 mF.cm^-2^ for open pore and approximately 180 mF.cm^-2^ for narrow 30-to-36-nm diameter PPy nanotube arrays attributed to intensive faradic processes arising from enhanced access of electrolyte ions through nanotube interior and exterior. Impedance spectroscopy studies show that capacitive response extends over larger frequency domain in electrodes with PPy nanotube structure. Simulation of Nyquist plots by electrical equivalent circuit modeling establishes that 3-D nanostructure is better represented by constant phase element which accounts for the inhomogeneous electrochemical redox processes. Charge-discharge studies at different current densities establish that kinetics of the redox process in PPy nanotube electrode is due to the limitation on electron transport rather than the diffusive process of electrolyte ions. The PPy nanotube electrodes show deep discharge capability with high coulomb efficiency and long-term charge-discharge cyclic studies show nondegrading performance of the specific areal capacitance tested for 5,000 cycles.

## Background

Electrochemical energy storage in the ultracapacitor devices is emerging as a frontline technology for high-power applications ranging from modern portable electronics to electric automotive. A battery-supercapacitor hybrid energy system is a power source that can meet the peak power demands in camera flashes, pulsed lasers, and computer systems back-up as well as electric propulsion in diverse industrial and vehicular transport applications. Among the materials systems, structured carbons which store charges as an electric double layer (EDL) in liquid electrolyte medium are widely studied with a focus on overcoming the energy-density limitation [1]. The materials systems which show capacitive function based on redox reactions are the insertion-type metal oxides and doped-conducting polymers capable of high energy-density storage [2,3]. The conducting polymers, such as polypyrrole (PPy), poly(3,4 ethylenedioxythiophene) (PEDOT), and polyaniline (PANI) which undergo redox processes equivalent of doping and dedoping of electrolyte ions as means of energy storage are being aggressively studied. These polymers exhibit pseudocapacitance properties due to presence of charge transfer reactions. The other most widely studied materials are the metal oxides RuO_2_, MnO_2_, V_2_O_5_, NiO, and Co_3_O_4_ which show highly capacitive behavior due to reversible and fast surface redox reactions with electrolyte ions [2,4].

In the recent years, conducting polymers with a nanoporous morphology and as nanocomposites with metal-oxides have emerged as the materials system of great potential for high energy-density storage. Electrodes based on these materials structured at the nanoscale enable many-fold enhancements of the electroactive surface and interface with electrolyte facilitating absorption, ingress, and diffusion of electrolyte ions which being the main energy storage units could lead to increased energy and power density of supercapacitor devices. The high surface area morphology in conducting polymers is attained by creating variations in its nanostructure like nanoporous [5], nanofibers [6,7], nanowires [8], nanobelts [9], and by size-selective nanopores in the context of carbons [10]. Most metal oxides are electrically resistive in character and the redox reactions here are limited to the surface regions. In order to accrue full advantage of their pseudocapacitive function, electrically conducting polypyrrole is encrusted by metal oxides [11,12], PEDOT [13], CNT [14], and graphene [15,16] in various nanostructured forms like nanoparticle [17,18], nanotube [19,20], and nanowire [21,22] as supercapacitor electrode. In these materials systems, the nanostructure features are randomly distributed in the two-dimensional (2-D) film form mainly due to the preparatory methods.

Most recent research thrust in the conducting polymers and their nanocomposite with metal oxides is directed towards the electrodes with three-dimensional (3-D) nanoarchitecture such as vertically aligned nanotubes [23] and nanorods [24]. These nanostructures have potential for the limiting electrolyte-ion diffusion problem by decreasing the ion diffusion paths and at the same time increasing the surface area for enhanced electrode-electrolyte interaction. In the past, randomly oriented conducting polymer nanotubes structures have been synthesized [16,25,26] for supercapacitor applications. However, the vertically oriented nanostructures, nanorods, and nanotubes have been mostly configured using the metal oxide templates [27]. Such nanostructures have been created by more innovating nanoscale engineering methods like oxidative polymerization [28], electrochemical anodic oxidation [29], electrodeposition [30], and hydrothermal synthesis [31,32]. Furthermore, by combining the redox conducting polymers with the well-known pseudocapacitive oxide like MnO_2_, forming the nanocomposites in the 3-D nanoarchitecture presents multiple advantages with enormous potential to outperform their 2-D counterparts. The composite 3-D nanostructure can be created by conformal deposition of redox-active conducting polymer, pseudocapacitive oxide layer, or their multilayer stacks over vertical nanostructures of TiO_2_, ZnO, or NiO serving as templates. The composite 3-D nanostructured electrodes have synergic contribution to specific capacitance based on their electroactive functions which boost energy density, and their nanoarchitecture have the ability to mitigate the ion diffusion limitation thereby enhancing the power density. In the past, 3-D nanotube polymers, PPy-PANI [33] polymer-metal oxides, TiO_2_-PPy [34,35], ZnO-PPy [36], TiO_2_-NiO [23], and TiO_2_-V_2_O_5_[37] have been reported.

In this work, we investigate the characteristics of nanocomposite electrodes for supercapacitors having 3-D nanoscale architecture, the one comprising of vertically aligned zinc oxide nanorod arrays at the core with doped-polypyrrole conducting polymer sheath and the other vertical polypyrrole nanotubes arrays. Although polypyrrole in the doped state shows high electrical conductivity, the conversion between redox states is very slow due to the slow transportation of counter ions to balance the charge in the polymer structure [38]. The vertical polypyrrole nanotube and sheath structure are likely to decrease the charge transfer reaction time and thus enhance the charge storage capabilities [38]. Zinc oxide used prominently in various devices such as biosensors [39], light emitting diodes [40], organic solar cells [41], and spintronics [42,43] is a biocompatible, highly stable, and less expensive material as compared to ruthenium oxide and therefore has a good potential for the electrochemical energy storage devices. Zinc oxide characterized as a wide band gap semiconductor with excellent chemical and physical properties can be easily transformed in various nanostructure forms like nanowire, nanoplatelets, and nanoneedles mostly as flat two-dimensional structures [44]. In the context of using a ZnO template for a supercapacitor electrode in the 3-D architecture, we have fabricated vertically aligned ZnO nanorods by hydrothermal synthesis which exhibit specific electrical and optical properties [32]. The nanocomposite electrode is formed by deposition of doped polypyrrole layer over ZnO nanorod at the core by the commercially viable, low cost solution-based pulsed current electropolymerization process [45]. Pulsed current process allows depositing polypyrrole layer selectively with highly controlled thickness through the application of number of pulses. Only a few studies have been reported on electrodes with zinc oxide and polypyrrole composite films for supercapacitor energy storage devices perhaps since zinc oxide nanostructures may be resistive compared to conducting polymers [46]. ZnO nanorods as template to create PPy nanotube structures with inlay of MnO_2_ and their energy storage behavior have been reported [36]. In this work, the conducting doped polypyrrole supercapacitor electrodes in two different 3-D architectural forms, one having ZnO nanorod core-PPy sheath and the other vertical PPy nanotube array have been investigated. The electrochemical properties of these electrodes were studied by impedance spectroscopy, cyclic voltammetry, and charge-discharge measurements. Randles circuit model with additional capacitance and resistance elements is developed to explain the characteristics of electrode at various frequency ranges. Long-term charge-discharge tests are carried out to evaluate the cycle life of such electrodes in supercapacitor energy storage device. This paper reports the results of these studies.

## Methods

### Synthesis of ZnO nanorod array template

Polypyrrole conducting polymer electrodes in the two ZnO core-PPy sheath and PPy nanotube structural forms were fabricated over a ZnO nanorod template. The template, a vertically oriented two-dimensional array of ZnO nanorods, was formed over surface-activated 500-μm-thin graphite substrates by thermo-decomposition of zinc nitrate aqueous solution in the presence of hexamethylenetetramine in a wet chemical process [32]. The surface of the graphite substrate is activated by depositing a 20-nm-thick ZnO seed layer that acts as nucleation centers for the growth of ZnO nanorods. This layer is formed by radio-frequency (RF) plasma sputtering from a stoichiometric ZnO target in the argon ambient at 50 mTorr pressure and 100-W RF power. The growth of ZnO nanorods is done in a solution of 0.03 M zinc nitrate hexahydrate (Sigma-Aldrich) and equimolar hexamethylenetetramine (HMT) (Sigma-Aldrich, St. Louis, MO, USA) in 18 MΩ deionized water. The surface-activated graphite substrate is vertically kept in this solution in the autoclavable glass bottle and held steady at 95°C for 8 hours. This hydrothermal procedure results in the dissociation of Zn(II)-amino complex resulting in ZnO which grows as ZnO nanorods [47]. Post deposition, the graphite substrates are rinsed in deionized water to remove any residual precursor chemicals and dried in air.

### Pulsed current electropolymerization of polypyrrole sheath and nanotube formation

A uniform and conformal deposition of polypyrrole of a controlled thickness over ZnO nanorods is essential for the creation of PPy sheath and nanotube nanostructures. Polypyrrole has been deposited the past by various chemical [48] and potentiostatic electropolymerization [49] methods. In this work, PPy deposition is done by electropolymerization of pyrrole monomer in situ over ZnO nanorods using the ultrashort multiple unipolar pulsed-current method reported earlier [45]. Electropolymerization is accomplished in an electrolyte solution containing 0.01 M pyrrole (Py) monomer (Sigma-Aldrich) and 0.1 M lithium perchlorate dopant ions in the presence of 0.06 M sodium dodecyl sulfate (SDS) surfactant. The preparatory steps involve first the dissolution of pyrrole monomer in the presence of SDS under continuous stirring in deionized water warmed to 40°C. Later, the ZnO-seed-layer-coated graphite substrate is dipped vertically for approximately 20 min in this solution which helps in the wettability of the ZnO nanorods with pyrrole monomer. Before initiating the electropolymerization process, lithium perchlorate is added to form 0.1 M solution. The formation of the polypyrrole sheath layer over ZnO nanorods is carried out by pulsed current electropolymerization method in a two-electrode cell using a platinum (Pt) sheet as a counter and a reference electrode. In this method, multiple unipolar anodic ultrashort 10-ms-duration constant-current pulses of amplitude 4 mA.cm^-2^ are applied. Each of the pulses is interspaced by a current ‘off’ period of 100-ms duration. The electropolymerization is initiated during the pulse ‘on’ period as the corresponding anodic potential exceeds the oxidation potential of the pyrrole monomer. The current off period essentially helps create the equilibrium conditions in the vicinity of ZnO nanorods for deposition under homogenous polymerization conditions. The number of current pulses effectively controls the polypyrrole-layer thickness and usually approximately 5 to 10 k pulses were used to form fully covered PPy sheath over ZnO nanorods. In some cases 20 k pulses were also applied to form a thicker PPy sheath.

The PPy nanotube structure is obtained by etching away the vertically aligned ZnO nanorod core in a 20% ammonia solution [36]. The ZnO etching process is typically initiated over ZnO nanorod tips due to relatively thin cladding of the electropolymerized polypyrrole. As a result, the PPy nanotube structure shows dependence on the etching time. In this work, etching times of 2 and 4 h are used for the formation of PPy nanotube arrays.

### Electrochemical characterization of supercapacitor electrodes

Efficacy of the ZnO nanorod core-PPy sheath and PPy nanotube electrodes for the supercapacitor energy storage device application was analyzed by various electrochemical characterizations. These electrodes were characterized by cyclic voltammetry (CV), alternating current (ac) impedance spectroscopy, galvanic charge-discharge, and long-term cyclic tests in a three-electrode cell with Pt sheet as counter electrode and the potential referenced to a saturated Ag/AgCl electrode in an electrolyte comprising of an aqueous solution of 1 M lithium perchlorate. Cyclic voltammetry and galvanic charge-discharge measurements were carried out using Solartron electrochemical interface (Model 1287 from Solartron Analytical, Oak Ridge, TN, USA). In cyclic voltammetry, the flow of electric current between the working electrode and Pt counter electrode was recorded in the potential range -0.5 to +0.5 V scanned at different rates between 5 to100 mV.s^-1^. The areal-specific capacitance, *C*_sv_ (F.cm^-2^), of the electrodes was calculated using the relation,

(1) $$C_{\mathrm{sv}}=\frac{i_{a}+\left|i_{c}\right|}{2s}$$

where *i*_a_ and *i*_c_ are the absolute values of the anodic and cathodic current (mA.cm^-2^) of the electrode area and *s* is the scan rate (mV.s^-1^). The galvanic charge-discharge characteristics were measured at various current densities, *i*_d_, varying between 1, 2, and 3 mA.cm^-2^ in the potential range of 0.05 to 0.5 V. In the discharge cycle, using the discharge time, Δ*t,* and a corresponding change in voltage, Δ*V*, excluding the IR voltage drop, the areal-specific capacitance *C*_sd_ (F.cm^-2^) is calculated by the relation,

(2) $$C_{\mathrm{sd}}=\frac{i_{d}\cdot \mathrm{\Delta t}}{\mathrm{\Delta V}}$$

The ac impedance measurements were carried out in a two-electrode configuration in the frequency range 1 mHz to 100 kHz with ac signal amplitude of 10 mV using Solartron Impedance/Gain-Phase Analyzer (Model 1260). Measured low-frequency imaginary impedance *Z*″ provides estimate of the overall capacitance *C*_i_ using the relation *C*_i_ = 1/|*ωZ*″|. The Nyquist plots using the impedance data were simulated using the equivalent electrical model representing the electrochemical and electrophysical attributes of the nanostructured ZnO-PPy electrode using ZPlot software (Scribner Associates, NC, USA) which provide the characteristic resistances and various contributing factors to the overall electrode capacitance.

## Results and discussion

### Microstructure of ZnO nanorod core-polypyrrole sheath, nanotube electrodes

**Figure 1:**
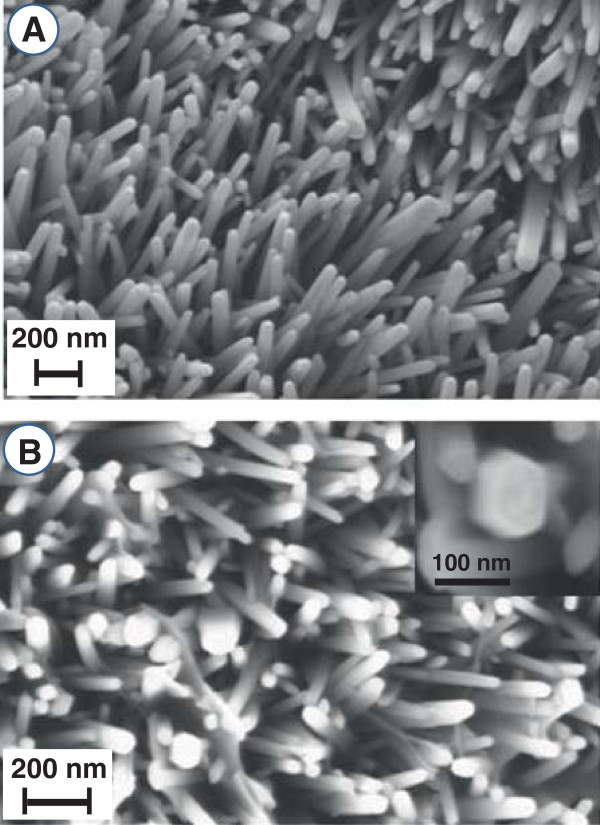
**SEM images of ZnO nanorod arrays grown on graphite substrate. (A)** Image showing the microstructure of ZnO nanorod arrays. **(B)** Magnified image showing the top end of the rods with hexagonal facets.

The microstructure of ZnO nanorod arrays grown over graphite substrates is shown by SEM micrograph in Figure 1A. These vertically grown ZnO nanorods are homogeneously dispersed across the substrate surface and their average length dependent on the growth time is typically approximately 2.2 to 2.5 μm. These rods appear to have smooth surface texture and nearly uniform average diameter of approximately 60 nm. As shown in the magnified image in Figure 1B and its inset, the top end of these rods have a hexagonal facet signifying these rods grow along the crystalline *c*-axis.

**Figure 2:**
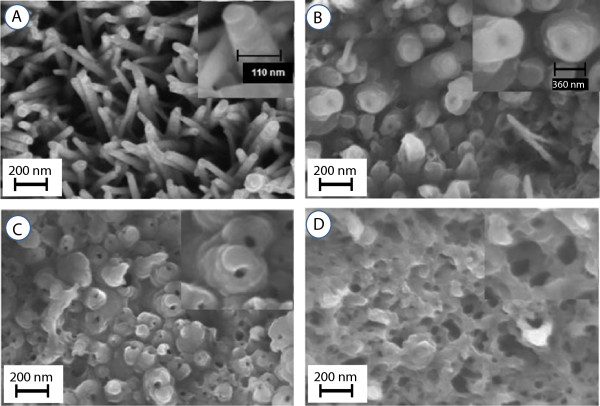
**SEM images of ZnO nanorod arrays coated with pulsed current polymerized PPy sheath. (A)** Initial stage of PPy oligomers cluster deposition, **(B)** ZnO core-PPy sheath structure after 10 k pulsed electropolymerization cycles, **(C)** PPy nanotube array after 2-h etch, and **(D)** open pore PPy nanotube array after 4-h etch.

**Figure 3:**
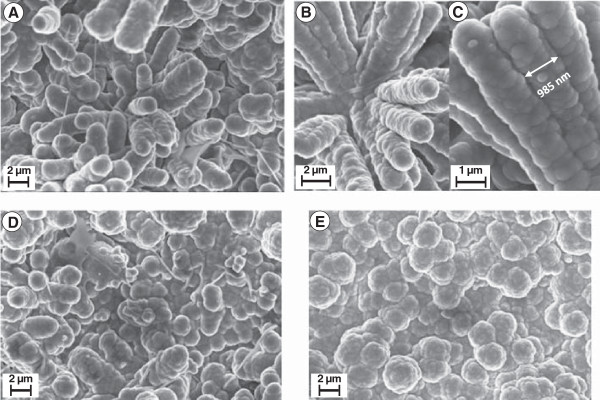
**SEM images. (A)** Thicker PPy deposited over ZnO nanorod array when electropolymerization was carried out for 20 k pulsed current cycles, **(B)** cross-sectional view of PPy sheath coated along the ZnO rod length, and **(C)** conjoined view of PPy sheath over ZnO nanorods with average diameter of 985 nm. Top view of ZnO nanorod tips with thick PPy sheath **(D)** before etch and **(E)** after ammonia etch.

**Figure 4:**
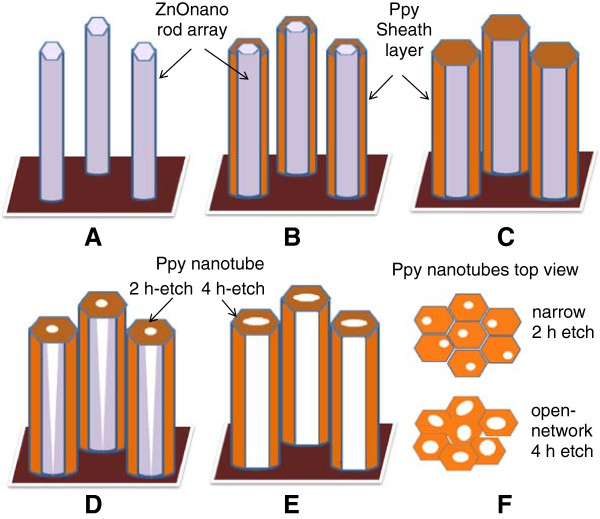
**Schematic representation of the evolution of the PPy sheath and nanotube structure.** Schematic representation of ZnO nanorod core coated by PPy sheath **(A-C)** and formation of PPy nanotube array after 2- and 4-h etch **(D-E)**. Top view **(F)**.

In the formation of PPy sheath over ZnO nanorods, its thickness is controlled by the number of pulsed current cycles. Figure 2A shows the early steps of the pulsed polymerization representing the formative stages of the growth of polypyrrole layer over ZnO nanorod arrays. It shows that the polypyrrole layer consisting of small compact nodular features forms conformal to the ZnO nanorods across its entire length. The nodular surface structure of polypyrrole layer is due to congregation of pyrrole monomer resulting from the action of SDS surfactant [50]. Furthermore, there is no deposition of polypyrrole in the interrod space and the PPy sheath forms preferentially over ZnO nanorods due to pyrrole monomer incursion by the action of the SDS surfactant as discussed later [50]. The inset shows a magnified view of a ZnO nanorod at the core coated with PPy sheath having overall average diameter of approximately 110 nm. Figure 2B shows ZnO core-PPy shell structure after electropolymerization has been accomplished for the full 10 k unipolar pulsed current cycles. The average diameter of the ZnO-core-PPy shell grows to approximately 360 nm which translates to approximately 150 nm average thickness of the PPy layer as shown by the magnified view of the top of ZnO nanorods in the inset of Figure 2B. At this growth stage, the inter-ZnO nanorod space begins to fill due to the coalescence of PPy sheath formed over different ZnO nanorods in the array. For the creation of the freestanding PPy nanotube array, the ZnO nanorod in the core is etched away in 20% ammonia solution. Figure 2C shows the partial etched state of the ZnO core for 2 h which creates tubular holes of approximately 30 to 36 nm in average diameter as shown in the inset of Figure 2C. At this stage, the PPy nanotube arrays still have in their interior a finite thickness of ZnO cladding. To remove the ZnO cladding, additional etching was carried out. It was observed that after a prolonged etching for approximately 4 h, a complete removal of the ZnO cladding was realized which resulted in the formation of a network of PPy nanotubular arrays as shown in the micrograph in Figure 2D. A magnified view in the inset shows PPy nanotubes of diameter approximately 60 to 70 nm consistent with the typical diameter of the ZnO nanorod core. Figure 2D also shows that a large number of these PPy nanotubes share a common sheath wall which had initially resulted from the PPy growth in the space between neighboring ZnO nanorods. This enables formation of the interconnected open network of freestanding PPy nanotube arrays. The PPy nanotube diameter can be enhanced by forming thicker ZnO nanorod array core structure. However, this reduces the effective thickness of PPy tubular sheath and hence the effective mass of PPy which is an active component for charge storage. On the other hand, increasing thickness of PPy by electropolymerization for longer pulsed current cycles excessively covers the top of the ZnO nanorod arrays making it difficult to etch away the ZnO core which prevented realization of PPy nanotubular arrays. Figure 3 shows the ZnO core-PPy sheath structure with the thicker PPy layer deposited using 20 k unipolar pulsed current cycles. This results in formation of thick conjoined PPy sheath with thickly deposited PPy over the top of ZnO nanorods (Figure 3A). Figure 3B shows a cross-sectional view indicating the ZnO nanorods could still be coated with PPy along its length. The side panel in Figure 3C shows conjoined PPy sheath over ZnO nanorods of average diameter approximately 985 nm to 1 μm. Morphology of the thick PPy deposit is like nodules. Figure 3D shows the top view of the PPy coated ZnO nanorods tips. Figure 3E shows the same view after ammonia etching for 4 h. It is evident that such ZnO nanorod core-PPy sheath structure did not result in the PPy nanotube structure after etching. The evolution of the PPy sheath and nanotube structure is schematically shown in Figures 4A, B, C, D, E, F. The vertical ZnO nanorod array (Figure 4A) is preferentially coated with PPy by pulsed electropolymerization process through surfactant action. Progressively, on continued pulsed current polymerization cycles, the PPy sheath thickness increases (Figure 4B) with possible merging of PPy sheath walls (Figure 4C). Figures 4D, E, F show the evolution of PPy nanotubes through etching of ZnO core starting at the nanorod tips which after short term etching results in the PPy nanotubes along with the inverted conical ZnO cladding (Figure 4D). The PPy nanotube arrays without the ZnO cladding are created by complete etching of ZnO for longer periods as depicted in Figure 4E with an open pore structure as shown in the top view in Figure 4F.

### Growth features of ZnO nanorod-PPy sheath and PPy nanotube arrays

**Figure 5:**
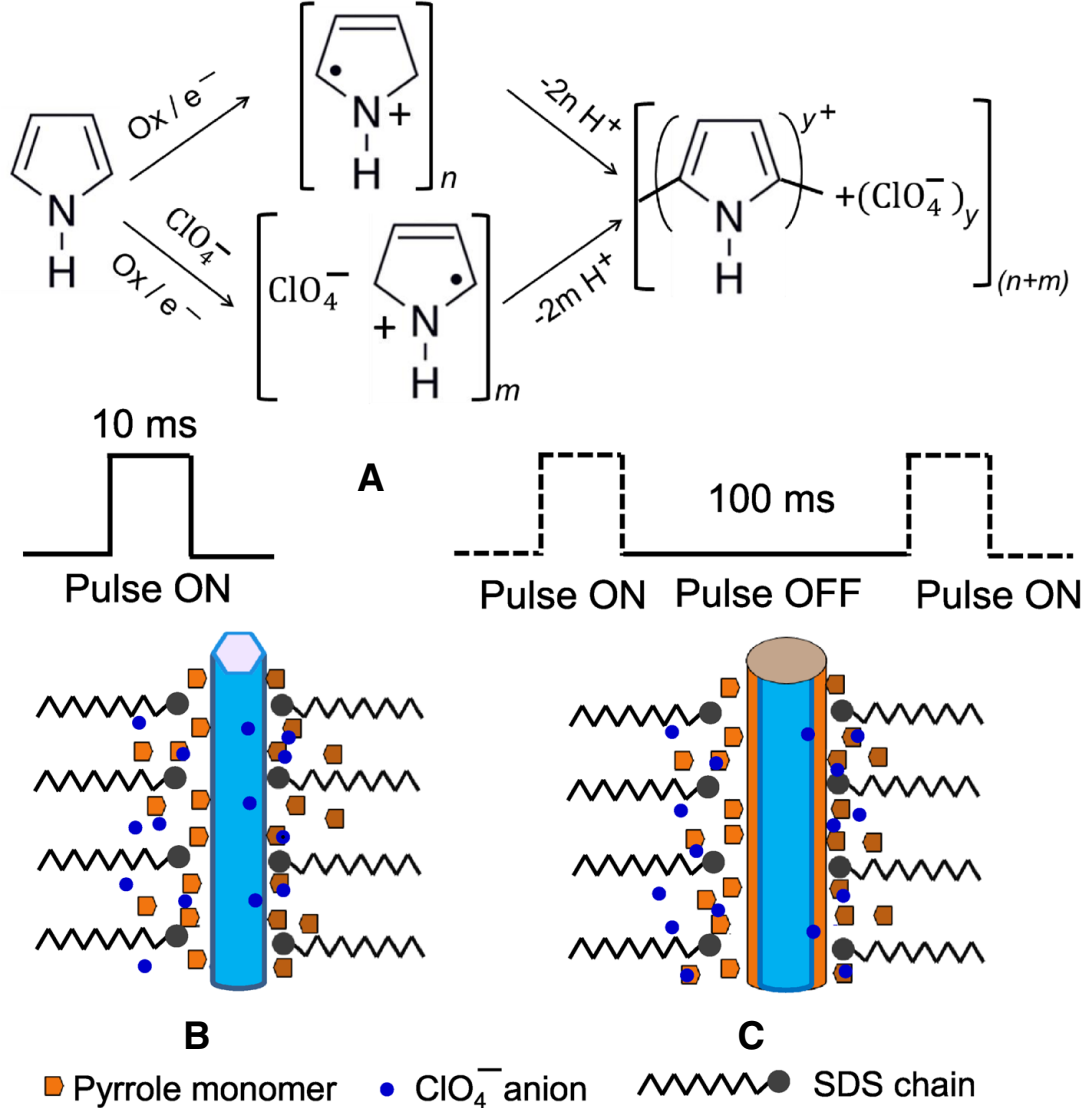
**Electropolymerization process of the polypyrrole growth over ZnO nanorods. (A)** Electrochemical polymerization of Py monomer and ClO_4_ conjugation. **(B)** Model of electropolymerization growth of PPy sheath over ZnO nanorods in the presence of SDS surfactant and **(C)** homogenous growth of PPy sheath over ZnO nanorods after a number of pulsed current cycles.

Unlike the two-dimensional flat conducting substrates in which case conventional direct current (dc) potentiostatic electropolymerization of pyrrole can produce uniform thick polypyrrole film, over the semiconducting ZnO nanostructures, pulsed current electropolymerization employed in this work was found essential to obtain homogeneous polypyrrole sheath. In order to create PPy 3-D tubular nanostructures for energy storage action, it is essential (i) to form the PPy sheath in the high-conductivity anion doped state and (ii) to have the PPy sheath of desired thickness coated uniformly over the entire length of the ZnO nanorod array at its core. The first criterion is largely met by anodic electropolymerization of pyrrole monomer in the aqueous medium in the presence of ClO_4_^2-^ anions derived from LiClO_4_ in the electrolyte. Various mechanisms of pyrrole electropolymerization have been proposed under potentiostatic condition [51,52]. In the pulsed current electropolymerization process, the polypyrrole growth over ZnO nanorod surface proceeds by concomitant reactions, anodic oxidation of pyrrole monomer, and conjugation reaction with electrolyte (ClO_4_^-^) anions as shown in Figure 5A. On application of a current pulse of magnitude 4 mA.cm^-2^, the pyrrole monomer species over the ZnO nanorod surface rapidly oxidize by electron transfer at electrode resulting in the nucleation of significantly large number of ${\left[\mathrm{Py}\right]}_{n+m}^{+●}$ cation radicals. By themselves, these are unstable but stabilize rapidly on interaction with the nearest cation radicals to form short chain oligomers by coupling and bond linkage with the involvement of deprotonation (-2H^+^)_*n*+*m*_ step [5,45,51,52]. A number of cation radicals ${\left[\mathrm{Py}\right]}_{m}^{+●}$ at the initiation step are also influenced by strongly interacting electrolyte ClO_4_^-^ anions which result in conjugation of PPy short chain oligomers deposited over ZnO nanorods [53]. The current pulse off time replenishes the Py-monomers at the ZnO nanorods by diffusion in the aqueous medium. The subsequent pulsed current cycle reinitiates the electropolymerization reaction at fresh nucleation sites on ZnO nanorods by a similar process sequence thus providing a uniform coverage.

The preferential nucleation and growth of polypyrrole across the ZnO nanorod length is significantly affected by the lack of access of the pyrrole monomer in deep crevices along the depth of ZnO nanorod array marked by the narrow and not-so-consistent interrod spacing typically varying between 120 to 250 nm. This is further aggravated by aqueous immiscibility of pyrrole monomer which inhibits wetting of ZnO rods which might inhibit formation of uniform polypyrrole sheath. In the present case, the use of SDS anionic surfactant mitigates this by transporting pyrrole monomer to the surface of ZnO nanorods. A possible model of electropolymerization growth of PPy sheath over ZnO nanorods in the presence of SDS surfactant is shown schematically in Figure 5B. The SDS ionizes into Na + cation and CH_3_(CH_2_)_11_OSO_3_^-^ anion in aqueous medium. The SDS concentration used in this study is less than the critical value 8 mM for the first micelles concentration (CMC-1) hence the SDS molecular chain containing 12 carbon alkyls with sulfate group at the end are in the extended state in the aqueous medium [54,55]. The dodecyl alkyl molecular chain being hydrophobic orients away from water and this easily attaches on to the ZnO nanorod surface while the hydrophilic OSO_3_^-^ group project outward into aqueous environment. The pyrrole monomers are hydrophobic in character and sparingly soluble in water. A large number of pyrrole monomers are able to preferentially disperse within the hydrophobic region created by attached dodecyl alkyl molecular chain over ZnO nanorod surface [50]. This ensures uninhibited supply of the pyrrole monomer and dopant ClO_4_^-^ anions across the exterior of ZnO nanorods [55] and consequently forming PPy layer over ZnO rods comprising of short-chain doped PPy oligomers by electronation-protonation-conjugation reaction described in Figure 5B. Spatially distributed deposition of PPy oligomers as clusters is evident in the nodule like the microstructure study shown in Figure 2A. The pyrrole monomer availability during current pulsed off time is no longer diffusion-rate limited and efficient incursion of pyrrole results in the increased electropolymerization rates. In the subsequent pulse cycles, the electropolymerization is reinitiated over new ZnO surface sites or over PPy coated surface as shown schematically in Figure 5C resulting in homogenous formation of the PPy sheath over ZnO nanorods after a certain number of current pulsed polymerization cycles.

### Cyclic voltammetry study

**Figure 6:**
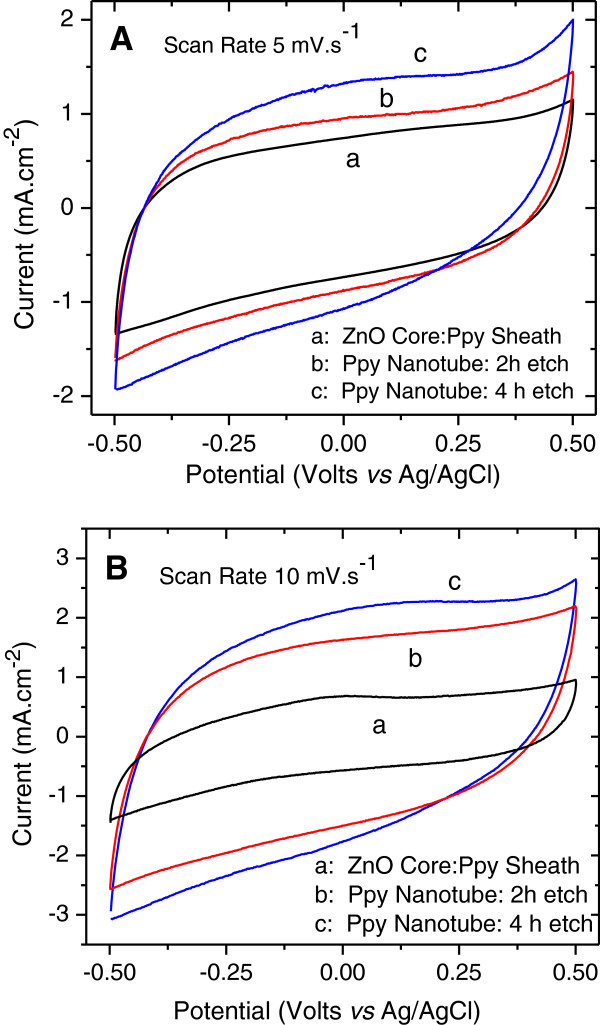
**Cyclic voltammetric plots of the electrode with nanostructured ZnO nanorod core-PPy sheath.** PPy nanotube after etching away ZnO nanorods for 2 and 4 h measured at scan rates **(A)** 5 mV.s^-1^ and **(B)** 10 mV.s^-1^.

**Figure 7:**
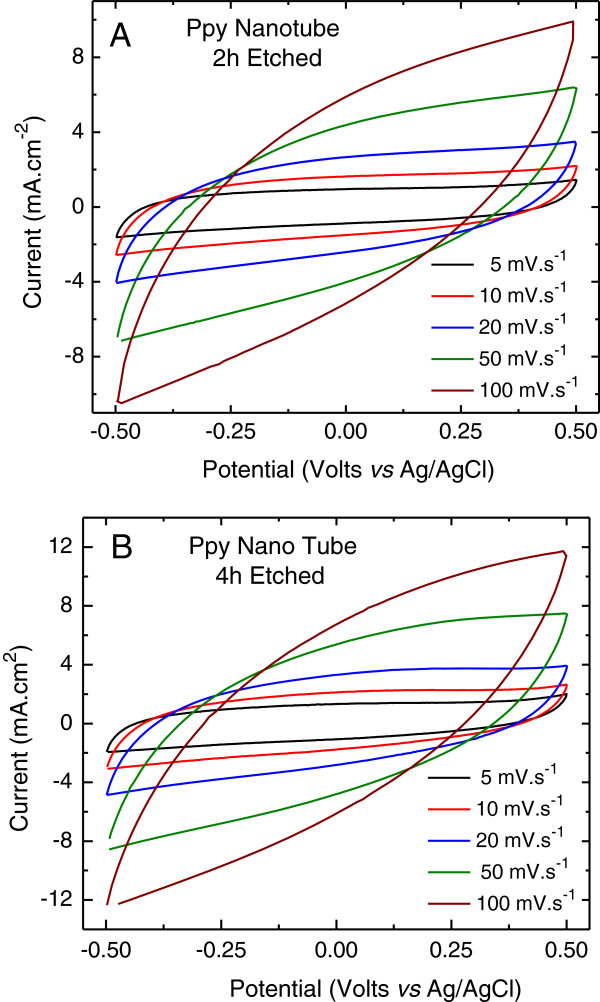
**Cyclic voltammetric plots of PPy nanotube electrodes measured at different scan rates. (A)** 2-h etch and **(B)** 4-h etch.

Figure 6A, B shows a set of CV plots recorded at slow scan rates of 5 and 10 mV.s^-1^ comparing the electrochemical performance of the ZnO nanorod core-PPy sheath electrode with the PPy nanotube structured electrodes obtained by etching ZnO nanorods for 2 and 4 h, respectively. All CV plots are nearly rectangular in shape, symmetrical across the zero current axis, and do not show any oxidation-reduction peaks demonstrating highly capacitive behavior. The current in the PPy nanotube electrodes is significantly higher in comparison to ZnO nanorod-PPy sheath electrode and it further increases for the electrode with an open pore PPy nanotube structure obtained after 4-h etching as shown by the microstructure in Figure 2D. It is apparent that PPy nanotube electrode structure offers improved access to the ions through the tube interior in addition to exterior regions which are accessed equally by all three electrodes. Figure 7A, B shows CV plots measured at scan rates of 5 to 100 mV.s^-1^ for the PPy nanotube electrodes obtained after etching of ZnO nanorods at the core for 2 and 4 h, respectively. The increase in the current with the scan rate indicates the kinetics of the faradic process and the electronic-ionic transport at the PPy nanotube-electrolyte interface. It is easy to observe from Figure 7 that more open PPy nanotube electrodes after 4-h ZnO etch show higher anodic and cathodic current at every scan rate as compared to the 2-h etched electrodes in the same potential window. Although both electrodes showed good charge propagation capabilities, the difference in the current density of the electrodes is attributed to the structural changes due to etching. The CV plots show that though rectangular shape is nearly preserved as the scan rate is increased until 50 mV.s^-1^, a general trend is a progressively narrower and slightly oblique-angled CV plot for scan rate of ≥50 mV.s^-1^. The factors responsible for such a behavior are the contact resistance and the delayed response time of the faradic reactions nonsynchronous with the faster scan which otherwise would have boosted the total capacitance.

**Figure 8:**
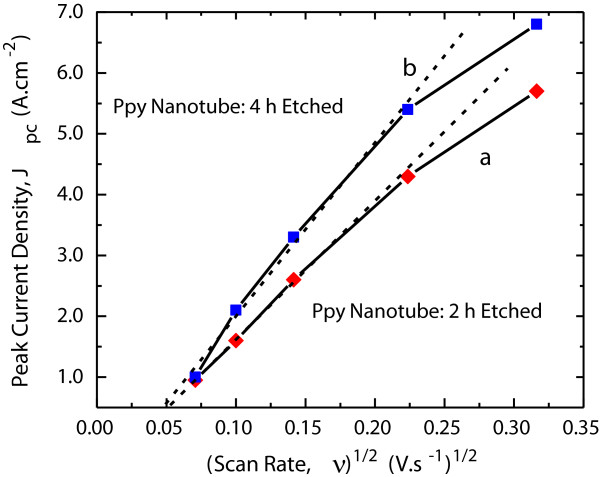
Randles-Sevcik plots of PPy nanotube electrodes after 2- and 4-h etching of ZnO nanorod core.

The growth in current density of the PPy nanotube electrodes with the increasing scan rate as shown in Figure 7 is reflective of the dissimilarities in terms of the porosity of the nanotube structure and improved performance of the 4-h etched PPy nanotube electrode. The rise in the cathodic peak current density *J*_PC_ with scan rate, *ν*, follows the Randles-Sevcik equation,

(3) $$J_{\mathrm{PC}}=0.6104n^{1.5}\mathrm{Fc}\sqrt{\frac{\mathrm{FD}}{\mathrm{RT}}}\cdot \sqrt{\nu \;}$$

where *F* is the Faraday number and *R* is the ideal gas constant. The active specie concentration in electrolyte is denoted by *c*, and the number of electron-involved reduction processes by *n*. The parameter *D* represents the apparent charge transfer coefficient by diffusion. A linear plot of the current $J_{\mathrm{PC}\;}\mathrm{vs}\;\sqrt{\nu \;}$ shown in Figure 8 for 2- and 4-h ZnO core etched PPy nanotube electrodes suggests that according to the Randles-Sevcik formulation the charge transport process is diffusive-controlled. Figure 8 shows that compared to the 2-h etched electrode, 4-h etched PPy nanotube electrode has a higher slope which suggests that in this electrode the electrolyte ions are more easily accessible due to the presence of higher diffusivity paths through interconnected nanotubes and therefore have improved ability to store charges. This is consistent with the microstructure study as well as the results of the CV analysis. The measured *D* values were found to be 7.27 × 10^-8^ and 1.09 × 10^-7^ cm^2^.s^-1^ for the PPy nanotube structure formed after 2- and 4-h etching, respectively, which is at least an order of magnitude higher than for the PPy films in 2-D porous structure [45]. These data show that homogenous transport dynamics of charge-compensating anions in the electrolyte is generally fast for 3-D PPy nanotubes especially for open interconnected PPy nanotubes formed after 4-h etch.

**Figure 9:**
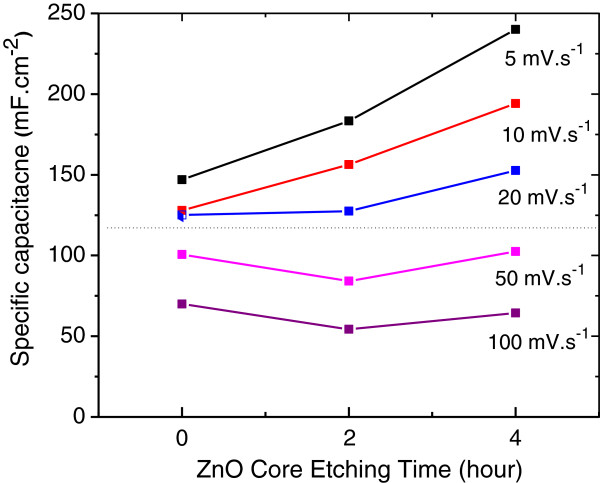
Specific areal capacitance at different scan rates for ZnO nanorod core-PPy sheath PPy and PPy nanotube electrodes.

Specific capacitance *C*_SV_ calculated from the CV plots using Equation 1 at different scan rates is plotted in Figure 9 for both ZnO nanorod core-PPy sheath and PPy nanotube electrodes represented by 0-, 2-, and 4-h ZnO core etch times. The true faradic specific capacitance due to redox processes measured at low scan rates increases dramatically when the PPy nanostructure transforms from core-sheath to nanotube. Thus, ion diffusion process in PPy nanotube structure is kinetically faster. At higher scan rates (≥50 mV.s^-1^), the specific capacitance on structure transformation shows moderate increase at best for electrode with open pore PPy nanotube structure obtained after a 4-h ZnO core etch. Limiting kinetics for ion diffusion is the same for PPy sheath and nanotube structures.

### Impedance spectroscopy

**Figure 10:**
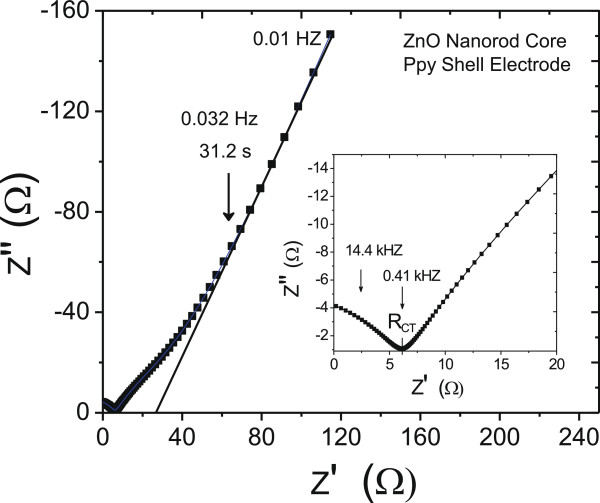
**Nyquist plots of actual data and fitted spectrum of ZnO nanorod core-PPy sheath electrode.** Inset shows expanded view in the high- and mid-frequency region.

Electrochemical impedance spectroscopy (EIS) technique is extensively used to elucidate the electrical characteristics of the electrode material and its interface with the supporting electrolyte. Frequency response of the real and imaginary impedance of the pseudocapacitive ZnO nanorod core-PPy sheath electrode with 1 M lithium perchlorate electrolyte was studied. Impedance of the electrode is a complex quantity and the extracted data are plotted as real (*Z*′) versus imaginary (*Z*″) impedance representing the Nyquist plot. Figure 10 shows the Nyquist plot of the as-deposited ZnO nanorod core-PPy sheath electrode in the frequency domain 0.1 MHz to 0.01 Hz and the inset shows expanded view in the high- and mid-frequency region. The capacitive component is reflected in the rapidly increasing imaginary impedance (*Z*″) at lower frequencies. The high-frequency real impedance (*Z*′) characterizes the bulk electrode and interfacial resistive properties of the electrode-electrolyte system. These parameters calculated from the impedance plots are shown in Table 1. Instead of the characteristic whole semicircle, the high-frequency Nyquist plot degenerated into an arc segment. This suggests that contribution to the bulk electrode-electrolyte resistance is mainly from the ZnO-PPy interface barrier due to polarization effect of the nanostructured electrode and negligible electrolyte resistance. The mid-frequency cutoff of this arc on real impedance *Z*′-axis gives the charge transfer resistance, *R*_CT_, of 6 Ω.cm^2^ resulting from the kinetically-controlled electron transfer and anion conjugation reaction in the PPy sheath layer. In progression from the mid- (0.41 kHz) to low-frequency range, a knee frequency of 0.032 Hz is identified indicating the onset of the capacitive impedance. The slow rising impedance in this frequency range is reflective of ion adsorption through the porous structure of the PPy sheath as well as along the length of ZnO nanorods. The capacitive impedance (*Z*″) shows a shift along more resistive *Z*′ values which is caused by the limitation on the rate of ion migration. Beyond the knee frequency, however, the system response is highly capacitive. The low-frequency areal-capacitance density, *C*_F_, is determined from the Nyquist plot as 107 mF.cm^-2^.

**Table 1:** Electrochemical impedance spectroscopy data obtained from actual Nyquist plots

**Components**	***R***_**s **_**(Ω *. *** **cm**^**2**^**)**	***R***_**ct **_**(Ω *. *** **cm**^**2**^**)**	***W *** **(Ω *. *** **cm**^**2**^**)**	***C***_**i **_**(mf.cm**^**-2**^**)**	***C***_**i **_**(f.g**^**-1**^**)**
ZnO nanorod core-PPy sheath	0	5.8	20.4	107.3	74
Narrow PPy nanotube (2-h etch)	0	8.2	8.4	84.2	58
Open PPy nanotube (4-h etch)	1	7.2	5.4	83	57.2

**Figure 11:**
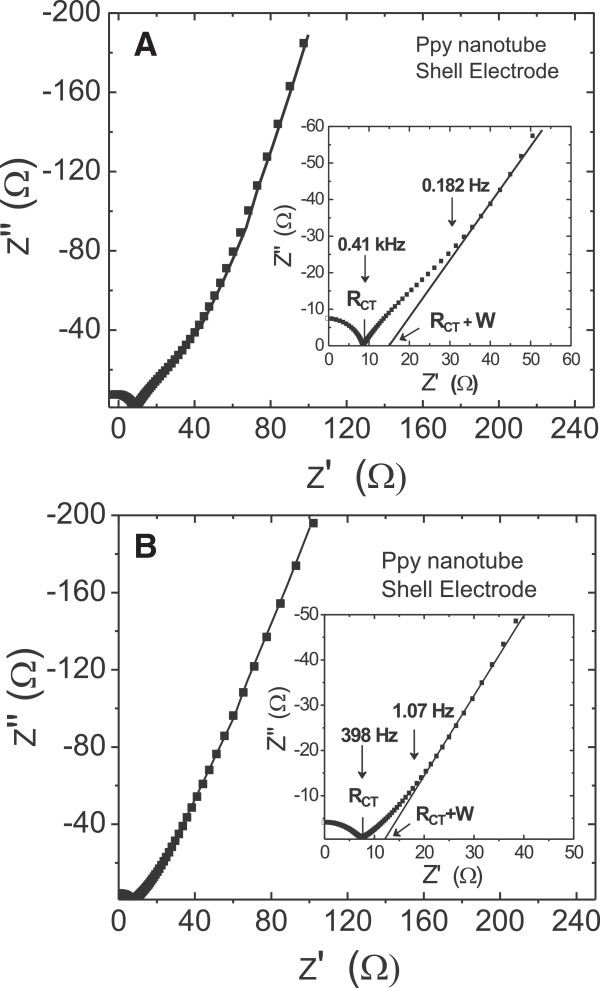
**Nyquist plots of actual data and fitted spectrum of PPy nanotube electrodes obtained after etching ZnO core. (A)** 2 h and **(B)** 4 h.

**Figure 12:**
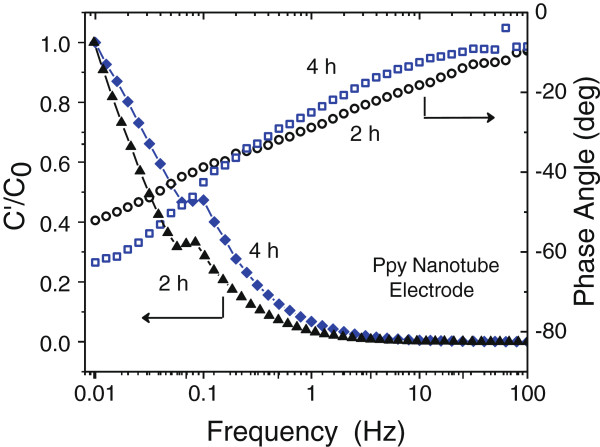
Frequency dependence of areal-specific capacitance to dc capacitance and phase angle variation for PPy nanotube electrodes.

Figure 11A, B shows the Nyquist plots of the PPy nanotube structure obtained after etching ZnO core for 2 and 4 h, respectively, as described by the SEM study in Figure 2C, D. The major effect of such structural change appears in the shift of the knee frequency to higher frequency values. After 2-h etching with narrow (33 ± 3 nm) PPy nanotube opening and after 4-h etching with open pore interconnected PPy nanotube formation the recorded shifts in knee frequency are 0.16 and 1.07 Hz, respectively, compared to the knee frequency of 0.032 Hz for unetched ZnO nanorod-PPy sheath structured electrode. This shift is significant. Simultaneously, the low-frequency impedance *Z*″ shows a systematic shift to lower values on the real impedance axis. Considering that knee frequency defines the upper frequency limit of the resistive behavior and a capacitive one at lower than knee frequencies, it is inferred that the PPy nanotube sheath structure is more capacitive in nature. Furthermore, for the unetched ZnO nanorod core-PPy sheath electrodes, the capacitance at knee the frequency is approximately 0.68*C*_F_ of the overall capacitance *C*_F_. Corresponding values for the 2- and 4-h etched PPy nanotube electrodes are 0.61*C*_F_ and 0.22*C*_F_, respectively. These data suggest that over a substantive frequency range the impedance of the PPy nanotube electrode is capacitive in nature. Clearly, the frequency domain of ion diffusion region which resistively contributes to impedance, commonly known as the Warburg resistance, has shrunk in PPy nanotubes after 2-h etching and more significantly in the open interconnected PPy nanotube structure obtained after 4-h etching of ZnO nanorods. It is obvious from the microstructure study of Figure 2C, D and schematic representation in Figure 4 that the interconnected network of 60 to 70 nm diameter open PPy nanotubes facilitates rapid and more pervasive ingression of anions from electrolyte as frequency decreases below the knee frequency. As frequency decreases, electrolyte ions by diffusion are accessible to more and deeper porous surface of the PPy nanotube arrays. The frequency response of the impedance is modeled in terms of complex capacitance *C*(*ω*) = *C*′(*ω*) - *jC*″(*ω*) to describe the capacitance behavior of the electrodes [56]. Here, *C*′(*ω*) is the real part of capacitance representing the energy storage component and *C*″(*ω*) the imaginary part represents the resistive losses in the storage process. The real capacitance is computed according to equation *C*′(*ω*) = [-*Z*″(*ω*)]/[*ω*|*Z*(*ω*)|^2^]. Figure 12 shows variation of *C*′/*C*_0_ as a function of frequency, where *C*_0_ is dc capacitance [57]. As the frequency decreases, *C*′ sharply increases below and above 1 Hz, the capacitance is practically nonexistent. Figure 12 also shows phase angle variation with frequency. The low-frequency phase angle shows a plateau at -65° for PPy nanotube sheath electrode after 4-h etching which indicates a capacitor-like behavior though not yet an ideal one for which phase angle should be closer to -90°. Compared to the nonplateau behavior and low phase angle of -40° observed in the unetched ZnO nanorod core-PPy sheath electrode, the PPy nanotube electrode shows considerably improved capacitor behavior.

The measured charge transfer resistance, *R*_CT_, is 8.2 and 7.2 Ω *cm*^2^, respectively, for 2- and 4-h etched PPy nanotube structured electrodes, which is not much different from that of the unetched ZnO nanorod core-PPy sheath structured electrode. It is obvious that extent of anion conjugation reaction in the PPy nanotube sheath in response to the electron transfer action is not much affected as the ZnO core is etched away. A more significant effect of the PPy nanotube sheath is seen in the Warburg impedance values. The intercept of extrapolation of the low-frequency impedance on the *x*-axis gives resistance *R*_CT_ + *W*, where *W* is the Warburg impedance. As shown in Table 1, *W* equals 20.2 Ω.cm^2^ for unetched ZnO nanorods core-PPy sheath electrode and decreases to 8.4 and 5.4 Ω.cm^2^ for the PPy nanotube structure realized after 2- and 4-h etching, respectively.

**Figure 13:**
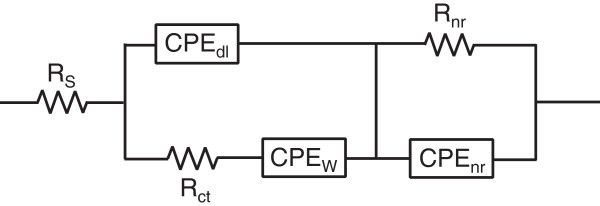
Equivalent electric circuit model used for simulation of Nyquist plots.

The impedance parameters of the complex ZnO nanorod core-PPy sheath electrode system were analyzed by equivalent circuit modeling. Nyquist plots are simulated using the equivalent circuit shown in Figure 13 and the component parameters were derived that provide closest fit at each frequency point [58]. Electrical equivalence of the physical electrochemical entities is marked in Figure 13. The equivalent circuit model includes solution resistance *R*_S_, charge transfer resistance *R*_CT_ representing the electrode kinetics, and Warburg element CPE_W_ representing the resistance encountered in diffusion and access of ions within nanoporous electrode structure. The inclusion of the constant phase element CPE_dl_ instead of the conventional purely capacitive element *C*_dl_ is to account for the dispersive behavior of the capacitance arising from the charge accumulation layer at the ZnO nanorods exposed to the electrolyte through pores in the PPy sheath and nanostructure of the electrode. Similarly, CPE_nr_ is the capacitive element which characterizes the pseudocapacitance property of the nanotubular PPy-anion conjugation. The nanostructure resistance, *R*_nr_, is representative of the electron transport resistance due to narrow (approximately 60 nm diameter) vertically long (approximately 2.2 μm) ZnO nanorods and *C*_nr_ its electrochemical capacitance [59]. The continuous lines in the Nyquist plots in Figures 10 and 11 are the results of the fitting based on this model. Excellent fit is observed over the entire frequency range. Various electrical resistance and capacitive parameters estimated by fitting of Nyquist plots are summarized in Table 2.

**Table 2:** Characteristic resistance and capacitive parameters estimated by fitting of Nyquist plots

**Components**	**CPE**_**dl **_**(mMho, p)**	***R***_**ct **_**(Ω)**	**CPE**_**w **_**(mMho, p)**	***R***_**nr **_**(Ω)**	**CPE**_**nr **_**(mMho, p)**
ZnO nanorod core-PPy sheath	*Q* = 0.025 *p* = 0.55	21.24	*Q* = 0.03 *p* = 0.61	6	*Q* = 0.012 *p* = 0.75
Narrow PPy nanotube (2-h etch)	*Q* = 0.0006 *p* = 0.87	18	*Q* = 0.036 *p* = 0.74	28	*Q* = 0.065 *p* = 0.44
Open PPy nanotube (4-h etch)	*Q* = 0.04 *p* = 0.61	16	*Q* = 0.04 *p* = 0.76	20	*Q* = 0.389 *p* = 0.42

**Figure 14:**
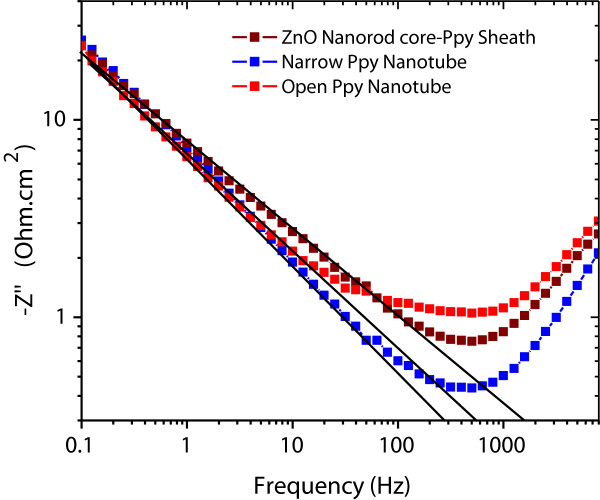
The log-log Bode plot of measured data for ZnO nanorod core-PPy sheath and PPy-nanotube electrodes at various frequencies.

The constant phase element (CPE) instead of the capacitor in the equivalent circuit above is justified in order to more appropriately account for the heterogeneities including the surface roughness, porosity, and variation in the PPy thickness arising from the nanostructured nature of the ZnO-PPy electrode. The long, vertical, and dispersed 3-D ZnO nanorod core-PPy sheath (nanotube) nanostructure has a diverse aspect ratio relative to a flat 2-D electrode structure and therefore differently impacts the ion diffusion kinetics. This gives rise to the distributed time constants simulating the capacitance dispersion which is better represented by the RC network comprising of nanostructure resistance, *R*_nr_, and the constant phase element, CPE_nr_[60]. The CPE_nr_ impedance is given as, [61].

(4) $$Z{\prime \prime }_{{\mathrm{CPE}}_{\mathrm{nr}}}=\frac{1}{{\left(\mathrm{j\omega}\right)}^{p}\cdot Q\;}$$

where exponent *p* represents dispersive nature of time constant, since with *p* = 1, the impedance *Z*″ is purely capacitive characterized by a single time constant and the parameter *Q* is equivalent to a capacitance, while for *p* < 1 parameter *Q* is basically a CPE with units Mho.cm^-2^. Figure 14 shows the log-log plot of measured *Z*″ versus frequency data for ZnO nanorod core-PPy sheath and PPy nanotube electrodes which is a straight line according to above Equation 4, and the slope corresponds to exponent, *p*. The *Z*″ calculated from the simulated exponent *p* and CPE *Q* values according to above equation and measured *Z*″ values are compared in Table 3. The low-frequency measured impedance, *Z*″, shows a deviation by a constant factor from the simulated CPE_nr_ impedance which is attributed to *C*_nr_ which has weak frequency dispersion but contributes to the measured *Z*″ values. The exponent *p* in the simulation of the impedance due to the Warburg element has the values 0.5 < *p* < 1 (Table 2). A *p* = 0.5 usually defines the CPE_W_ due to Warburg impedance. The higher simulated *p* values are interpreted as diffusion-based resistance signifying the pseudocapacitive nature of electrodes.

**Table 3:** **The *Z*** ″ **parameter calculated from the simulated exponent *p *** **and CPE *Q *** **values and the measured *Z*** ″ **values**

**Nanostructure electrode**	**Simulated** $Z'{'}_{{\mathrm{CPE}}_{\mathrm{nr}}}\mathrm{mhos}.{\mathrm{cm}}^{-2}s^{-p}$	**Measured** $Z'{'}_{{\mathrm{CPE}}_{\mathrm{nr}}}\mathrm{mhos}.{\mathrm{cm}}^{-2}s^{-p}$	**Percentage deviation**
ZnO nanorod core-PPy sheath	11.8	7.7	34.7
Narrow PPy nanotube (2-h etch)	11.2	7.15	36.1
Open PPy nanotube (4-h etch)	9.59	6.50	32.2

### Charge-discharge curves and stability analysis

**Figure 15:**
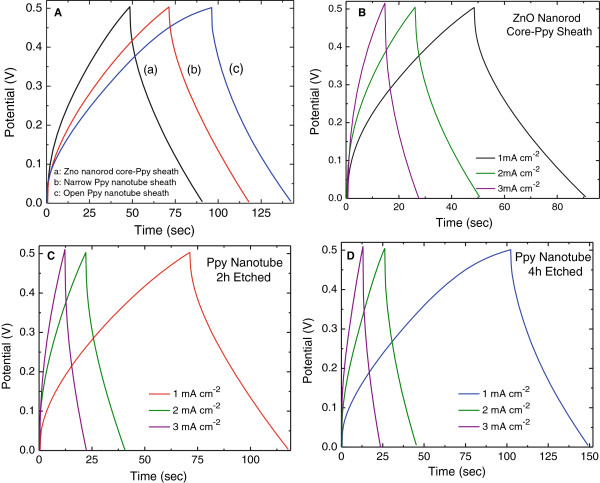
**Charge-discharge characteristics. (A)** ZnO nanorod core PPy sheath electrode and PPy nanotube electrodes after 2-h and 4-h etch measured at a constant current density of 1 mA.cm^-2^. Charge-discharge characteristics measured at different current densities for **(B)** ZnO nanorod core-PPy sheath, **(C)** PPy nanotube 2-h etch, and **(D)** PPy nanotube 4-h etch.

**Figure 16:**
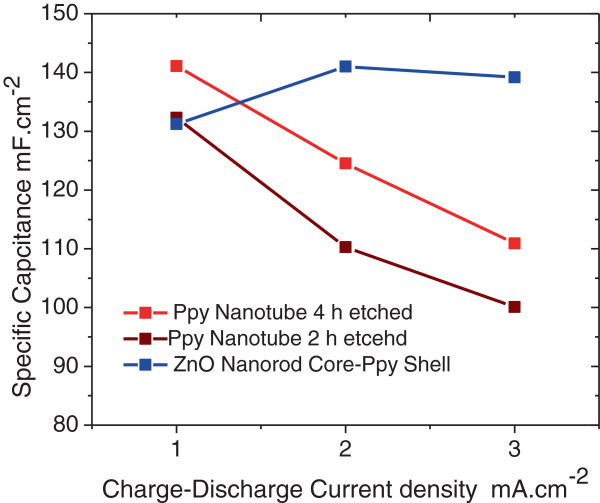
The specific capacitances of the ZnO nanostructured electrodes plotted as a function of charge-discharge current density.

Galvanostatic charge-discharge performance of the ZnO nanorod core-PPy sheath and PPy nanotube electrodes was studied at various current densities from 1 to 3 mA.cm^-2^ in the voltage range 0.05 to 0.5 V. Figure 15A shows charge-discharge curves measured at 1 mA.cm^-2^ for the PPy nanotube electrode obtained by a 2- and 4-h etch and its comparison with that of the ZnO nanorod core-PPy sheath electrode. The discharge curves are nearly linear for all the three electrode structures indicating highly capacitive character. The areal-capacitance density *C*_sd_ of the electrodes was calculated from the charge-discharge curves at 1 mA.cm^-2^ using Equation 2 and the results are presented in Table 4. The electrode with PPy open nanotube structure is higher than the electrode with ZnO nanorod core-PPy sheath structure. This suggests that electrolyte ions are able to access through nanotubes and can intercalate better with PPy taking advantage of the exposed nanotube surface. A small IR drop at the start of the discharge cycle is noticed in each of these electrodes which is due to equivalent series resistance (ESR) arising from contacts and internal electrode cell resistance. Typical ESR values are in the range 25 to 40 Ω.cm^2^ as shown in Table 4. The internal resistance contribution to the ESR originates from the core and the thin ZnO seed layer at the graphite substrate. In the charge (doping) cycle extraction and in the dedoping (discharge) cycle, the ingress of electrons from and into the PPy tubular shell has a percolation path through ZnO rods which offers a finite resistance. This is evident from the significantly lower ESR values in the electrodes with the partial dissolution of ZnO core with narrow opened PPy nanotubes obtained after a 2-h etch. A slight increase ESR is observed in the electrodes with open PPy nanotube structure. The contact between PPy sheath over ZnO nanorods and the others in the vicinity is minimal at best as the sheath thickness is on the average less than the inter-ZnO nanorod spacing (see Figures 2, 3 and 4). After complete dissolution of ZnO, the finite contact resistance between the freestanding PPy nanotube sheaths is responsible for increase in ESR. The effect of charge-discharge current density on the charge-discharge characteristics for each of these electrodes in ZnO nanorod core-PPy sheath PPy nanotube structures is shown in Figure 15B, C, D which follows a similar trend as discussed in the context of Figure 15A. The specific capacitances of these electrodes were calculated at different constant current density and the results are plotted in Figure 16 as a function of discharge current density. In the case of PPy nanotube electrodes, a decrease in the specific capacitance with increasing discharge current is observed. This suggests that the redox process is kinetically dependent on the ionic diffusion at the PPy nanotube-electrolyte interface even though the nanotubes have an unabated access to the ions as evident from the increased specific capacitance of the electrode with open PPy nanotube structure over the one having narrow PPy nanotube structure. The nearly constant specific capacitance of the ZnO nanorod core-PPy sheath electrode with increasing discharge current density is suggestive of faster redox kinetics at the interface. These observations suggest that the redox process in the PPy nanotube electrodes is due to limitation on electron transport rather than the diffusive access of electrolyte dopant ions to the PPy in the nanotube structure. The electron transport is facilitated through ZnO nanorods in close contact with graphite substrates. In the case of PPy nanotubes, electron transport can only take place through the PPy nanotube along its length. Since anion conjugation (doping) is in response to the electron extraction in spite of unimpeded access to electrolyte anions, the doping process is limited by electron transport. The reduction in the specific capacitance in PPy nanotubes at higher charge current and the increase in specific capacitance of 3-D ZnO nanorod PPy sheath structure electrode with the increase in charging current as observed in Figure 16 are explicable on this basis.

**Table 4:** **Specific areal capacitance and ESR of nanostructured electrodes calculated from charge-discharge curves measured at 1 mA.cm**^**-2**^

**Nanostructure electrode**	***C***_**sd **_**(mF.cm**^**-2**^**)**	**ESR (Ω.cm**^**2**^**)**
ZnO nanorod core-PPy sheath	131.22	40.5
Narrow PPy nanotube (2-h etch)	132.28	25.08
Open PPy nanotube (4-h etch)	141.09	32.09

### Cycling test

**Figure 17:**
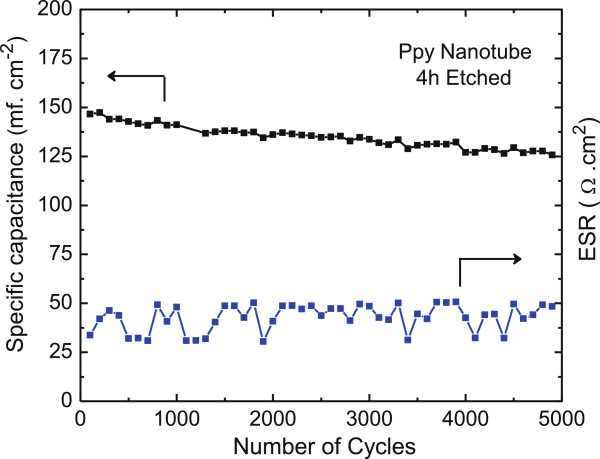
Long-term charge-discharge cycle tests for PPy nanotube 4-h etched electrode showing discharge capacitance density and ESR variation.

The cycling stability of the open PPy nanotube electrode was investigated at a constant charge-discharge current density of 1 mA.cm^-2^ for a continuous 5,000 cycles. Figure 17 shows the effect on the discharge capacitance density as a function of the number of charge-discharge cycle. The overall change in the discharge capacitance is only <12% indicative of highly stable redox performance and electrochemical stability of the PPy nanotube electrode. This stability arises from unimpeded access of the electrolyte ions through diffusive transport across to a large fraction of the PPy polymer surface due to the 3-D nanotube structure in the redox process. Furthermore, the PPy nanotube electrodes do not show physical or chemical degradation during cycling. This is borne out from the ESR data, which remains on the average nearly constant during cycling tests for 5,000 cycles.

## Conclusions

Electrodes in the three-dimensional nanoscale architecture studied in this work in the form of vertically aligned ZnO nanorod PPy sheath and PPy nanotube show considerable potential for high energy-density storage in a supercapacitor device. These nanostructures are formed by depositing a sheath of PPy over vertical ZnO nanorod arrays by controlled pulsed current electropolymerization and by selective etching of the ZnO nanorod core. Based on the cyclic voltammetry data, electrode with open interconnected PPy nanotube array structure shows high areal-specific capacitance of approximately 240 mF.cm^-2^ attributed to realization of enhanced access to electrolyte ions. The observed scan rate dependence of the current has been interpreted as delayed response time of faradic reaction nonsynchronous with faster scan rate, which could possibly have boosted capacitance density further. Slow redox processes are shown to be due to limitation of electron transfer across the length of vertical PPy nanotube arrays rather than the diffusive transport of electrolyte ions. Managing this limitation could possibly enhance the specific capacitance and thus energy storage ability further.

## Competing interests

The authors declare that they have no competing interests.

## Authors' contributions

NKS carried out the experiment and data analysis. ACR guided the study and helped in data interpretation. Both authors read and approved the final manuscript.

## Authors' information

NKS is presently a PhD student at the Electrical and Computer Engineering Department at the State University of New York, Binghamton. ACR is Associate Professor at the Electrical and Computer Engineering Department and Associate Director of the Center for Autonomous Solar Power (CASP) at the State University of New York, Binghamton.
